# A Company Is Only as Healthy as Its Workers: A 6-Month Metabolic Health Management Pilot Program Improves Employee Health and Contributes to Cost Savings

**DOI:** 10.3390/metabo12090848

**Published:** 2022-09-09

**Authors:** Nicholas G. Norwitz, Adrian Soto-Mota, Tro Kalayjian

**Affiliations:** 1 Harvard Medical School, Boston, MA 02115, USA; 2 Metabolic Diseases Research Unit, National Institute for Medical Sciences and Nutrition Salvador Zubiran, Tlalpan, CDMX 14080, Mexico; 3 Monterrey Institute of Technology and Higher Education, School of Medicine, CDMX 14380, Mexico; 4 Yale New Haven Health System, New Haven, CT 06830, USA

**Keywords:** carbohydrate restriction, diabetes, metabolic health, obesity, telemedicine

## Abstract

Chronic diet-related metabolic diseases, including diabetes and obesity, impose enormous burdens on patient wellness, healthcare costs, and worker productivity. Given the interdependent nature of the human and economic costs of metabolic disease, companies should be incentivized to invest in the health of their workforce. We report data from an ongoing pilot program in which employees of a manufacturing company with obesity, prediabetes, or diabetes are being treated by a metabolic health clinic using a carbohydrate restriction, community-orientated telemedicine approach. 10 patients completed the first 6 months of the program, and all lost weight, with a mean weight reduction of 38.4 lbs (17.4 kg). Improvements in HbA1c, fasting glucose, HOMA-IR, triglycerides, C-reactive protein, and systolic blood pressure were also observed across the group. Furthermore, the 10-year risk of having a major cardiovascular event, as calculated by the American Heart Association risk calculator, decreased from a mean of 9.22 to 5.18%, representing a 44% relative risk reduction. As a result of improvements in their metabolic health, patients were able to discontinue medications, leading to an estimated annualized cost savings of USD 45,171.70. These preliminary data provide proof-of-principle that when companies invest in the metabolic health of their workers, both parties stand to gain.

## 1. Introduction

The human and economic burdens of chronic diet-related metabolic diseases are increasing in severity. Diabetes provides an illustrative case in point. According to the Centers for Disease Control and Prevention, 1 in 10 Americans have diabetes, and 1 in 3 have prediabetes. One in four healthcare dollars is currently spent on diabetes, amounting to expenses of greater than USD 1 billion per day across the United States [1,2]. As the human burden rises, projected to reach one-third of Americans given the current trajectory, so will the economic costs, leading to a situation that is both abhorrent from a humanitarian standpoint and non-viable from an economic one.

Not only do diet-related metabolic diseases negatively impact the person afflicted with the condition directly, but there are further negative effects on work efficiency. A study conducted by the American Diabetes Association in 2017 estimated that the indirect cost of diabetes alone amounts to USD 90 billion in terms of reduced productivity [3], and it is unlikely that these figures will improve in the ensuing years. Similarly, there is extensive literature demonstrating that obesity imposes both an enormous human burden and exerts a toll on worker productivity [4,5,6].

The interdependence of employee health and work productivity provides both ethical and financial incentives for companies investing in employee wellness. Herein, we report retrospectively on the results from the first 24 weeks of an ongoing metabolic health management pilot program, conducted as a partnership between a manufacturing company and a medical weight loss and metabolic health clinic. The clinical aim of the project was to use a patient-centered individualized low-carbohydrate lifestyle approach to improve the metabolic health, including weight loss and improvements in cardiovascular risk scores, of patient-employees living with obesity, prediabetes, or diabetes. Additionally, reductions in health care costs were achieved through deprescription.

## 2. Materials and Methods

### 2.1. Establishment of an Employee Metabolic Health Management Program

A self-insured manufacturing company approached the Medical Weight Loss clinic of a co-author, Dr. Tro Kalayjian (TK), to establish an employee metabolic wellness program in October 2021. Between October and December, the company advertised the program through internal posters, fliers, and emails. The clinic held an information session for the company in December. Thereafter, employees were selected (described below) and established care with the clinic in December 2021. The clinic was compensated per standard patient monthly fee.

### 2.2. Patient Enrollment, Consent, and Completion

12 patients were initially selected based on clinical judgment of overall medical need, as determined by TK following assessment of the severity of obesity, diabetes status (HbA1c), and medication burden. All patients were initially onboarded purely with clinical intent (details below). Their intent was to only publish data following the completion of the first 24 weeks of the program; at this time, all patients provided further written informed consent to have their deidentified data analyzed and published. Yale New Haven Health Bridgeport Hospital IRB (#082225) granted exemption as this work constitutes secondary research data.

After the first 12-week quarter of the program, two patients discontinued, with one citing family issues and the other becoming ineligible due to termination of their employment at the manufacturing company, leaving 10 who completed the first 24 weeks.

### 2.3. In-Clinic Baseline and Quarterly Assessments

Blood tests for all patients at baseline (0 weeks), at the end of the first 12-week quarter (Q1) and at 24 weeks at the end of the second quarter (Q2), were collected after a 12 h overnight fast and included a complete blood count, comprehensive metabolic panel, vitamin B12, folate, C-reactive protein (CRP), HbA1c, and lipid panel.

### 2.4. At-Home Monitoring

For the purposes of daily use, at-home self-monitoring, and biofeedback, patients were provided Freestyle Libre 2 continuous glucose monitors (CGMs) (Abbott, Abbott Park, Chicago, IL, USA), glucose–ketone meters (Keto Mojo, Nappa, CA, USA), body weight scales, and sphygmomanometers (Qardio, San Francisco, CA, USA).

### 2.5. Team-Based, Patient-Centered, Asynchronous, Community-Orientated Medicine

The care team for this metabolic health management pilot program included TK, two medical assistants, two health coaches, and one personal trainer. Each patient met with a medical assistant for 30 min to receive an overview of the program, assist with the implementation of their equipment, and begin using the practice’s mobile application.

During the initial 90 min onboarding appointment, TK and a health coach reviewed the patient’s prior lab results, medical history, personal goals for the program, and food restrictions and preferences. Thereafter, patients attended weekly virtual meetings with TK or a health coach. Additionally, patients were encouraged to contact the care team via a secure messaging platform (Spruce) with personal medical or nutritional questions. Patients had access to real-time biofeedback vis-a-vis their CGM readings and were encouraged to explore the clinic’s app for asynchronous virtual support and education. The app focuses on the science of hunger, appetite, and cravings, as well as the role of stress and sleep hygiene on health and well-being. Patients were encouraged to use the app, where they had access to informational articles on nutrition and metabolism, live streams and group meetings with health coaches, as well as a monitored community group chat devoted to the company’s employees.

In terms of specific dietary advice, patients were all initially recommended some variation of a ketogenic diet. The specific dietary composition was designed individually based on the patient’s allergies, food aversions, and taste preferences. However, all patients were instructed to restrict net carbohydrates (total carbohydrates minus fiber) to fewer than 30 g to maintain nutritional ketosis; in this context, patients were also encouraged to eat according to their subjective hunger and experiment with intermittent fasting patterns, such as an eight or six hour feeding window.

Holistically, the aim of the program was to empower patients with information about how to reach their health goals and then nurture their personal growth with access to self-monitoring tools and a supportive healthcare community. The program included nearly daily touch points through equipment use, the clinic’s app, and/or direct secure messaging with the clinic team members.

### 2.6. Statistics

This retrospective chart review was conducted on a convenience sample; thus, power analyses were not performed prior to patient enrollment. As noted above, two patients discontinued the program after the first quarter, with one citing family issues and the other becoming ineligible due to termination of their employment at the manufacturing company, leaving 10 who completed the first 24 weeks of the program. One of these 10 had type 1 diabetes, was on a closed-loop system, and was excluded from analyses a priori as their distinct metabolic condition would bias the data for the rest of the cohort.

Data management and statistical analyses were performed using R version 4.0.3 (with R Studio version: 1.3.1093), and all of the R packages were updated to their latest version (30 July 2022). Descriptive results were produced with the function *tableone::CreateTableOne*. The statistical significance threshold was established at 0.05 and evaluated by a Friedman’s test. Post-hoc pair-wise tests were performed using a Durbin–Conover test.

Comparisons, including distribution curves and violin box plots (Supplementary Materials Figure S1), were created using the function *ggstatsplot::ggwithinstats,* which automatically provides both violin box plots and confidence intervals with effect size calculations [7]. Default parameters for non-parametric tests were applied as the data were non-Gaussian in distribution.

The anonymized raw data from the step-by-step commented code for reproducing both quantitative and graphical analyses are publicly available at: https://github.com/leaduiem/10yrcvdrisk (accessed on 15 July 2022).

The 10-year risk of a major cardiovascular event for each patient was calculated using the 2019 version of the American College of Cardiology (ACC) and American Heart Association (AHA) risk calculator (https://www.cvriskcalculator.com (accessed on 15 July 2022)), updated from the initial 2013 ACC/AHA guidelines [8]. In those instances where patients had total cholesterol or LDL-C levels below the 130 and 70 mg/dL minimum thresholds required to use the calculator, values of 130 and 70 mg/dL, were entered.

Drug costs were estimated using those listed on GoodRx (GoodRx.com (accessed on 15 July 2022)).

## 3. Results

Statistics were performed on the data from 9 of the 12 initial patients. Two patients discontinued after the first 12-week quarter, and data from the single patient with type 1 diabetes was isolated a prior as they were on a closed-loop system. These three trends tended to follow those of the other nine patients, as specified in the text below.

### 3.1. Baseline Characteristics

The patients’ mean age was 52.9 ± 5.7 years, 56% were female, and all were non-smokers. As displayed in Table 1, the mean starting weight, BMI, and HbA1c were 290.5 ± 44.9 lbs (131.8 ± 20.4 kg), 48.3 ± 6.8 kg/m^2^, and 7.1 ± 1.4%, respectively. All patients who enrolled at 24 weeks had prediabetes or diabetes at baseline.

### 3.2. Main Results

**Figure 1:**
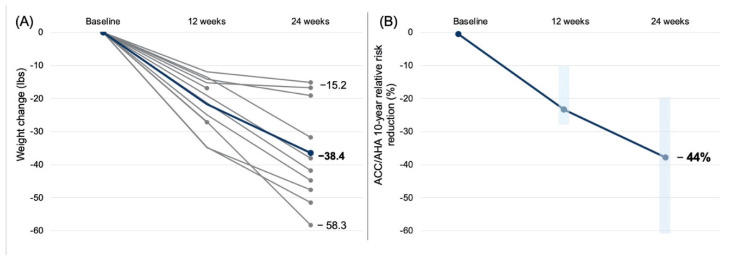
(**A**) Individual patient weight loss data for all 12 patients. Mean weight loss was 38.4 lbs (17.4 kg) among the 10 patients enrolled at 24 weeks, with a range of 15.2 to 58.3 lbs (6.9 to 26.4 kg) lost. All patients lost weight. (**B**) Reduction in the 10-year risk of experiencing a major adverse cardiovascular event as determined by the American College of Cardiology and American Heart Association calculator. The mean relative risk reduction was 44%, corresponding to an absolute mean risk reduction of 4.04%. Vertical light blue bars represent the 25th to 75th percentile changes derived from the violin box-and-whisker plot provided in Supplementary Materials Figure S1.

The mean weight loss was 38.4 lbs (17.4 kg), with a range of 15.2 to 58.3 lbs (6.9 to 26.4 kg) lost (Figure 1A). The patient with type 1 diabetes similarly lost 19.1 lbs (8.7 kg), and those who discontinued after the first quarter lost 27.1 and 16.9 lbs (12.3 and 7.7 kg); thus, all patients lost weight. The HbA1c reduced by 1.1% (Table 1). The two patients with the highest starting HbA1c (10.3 and 7.7%) also exhibited the largest decreases in HbA1c (−4.4% and −1.9%, respectively). Across the group, in addition to significant decreases in weight, BMI, and HbA1c, there were significant decreases in HOMA-IR (from median 11.1 to 2.2), CRP (median 9.3 to 8.1 mg/dL), and systolic blood pressure (median 140 to 121 mmHg) over the course of the 24 weeks. Further statistical details are provided in Supplementary Materials Figure S1.

Importantly, we observed a significant 4.04% absolute reduction in the 10-year risk of major adverse cardiovascular events (from 9.22 to 5.18%). As patients had highly heterogeneous baseline risks (ranging from 2.0 to 28.4%), we also reported a mean relative risk reduction of 44% in the 10-year ACC/AHA score (Figure 1B). Notably, these values for risk reduction may underestimate the magnitude of cardiovascular disease risk reduction as several patients exhibited total cholesterol and LDL-C values below the minimum threshold for using the ACC/AHA calculator and experienced improvements in metabolic health (CRP, HbA1c, HOMA-IR) not captured by this score.

### 3.3. Deprescription and Estimated Cost Savings

Concurrent with improvements in metabolic health, as described above, many patients were able to reduce or eliminate certain medications, including those for glycemic control, blood pressure, and lipid lowering. No patients were started on new medications. Based on the listed commercial prices of deprescribed medications, cost savings across the cohort over 24 weeks were calculated and projected to annualized savings of USD 45,171.70 (Supplementary Materials Table S1).

## 4. Discussion

We showed that when an independently insured manufacturing company pursued a telemedicine metabolic health/carbohydrate restriction program for its employees, employee health improved substantially—including reductions in weight, HbA1c, CRP, and 10-year ACC/AHA risk score—along with a reduction in estimated medication costs. There is a wide and growing body of evidence suggesting that carbohydrate-restricted diets may hold particular benefits for those living with metabolic conditions such as obesity, diabetes, and metabolic syndrome [9,10,11,12,13,14]. There is also prior data from a cohort in the United Kingdom showing that educating patients with type 2 diabetes on the glycemic and metabolic benefits of carbohydrate restriction can help reduce HbA1c alongside deprescription, leading to cost savings within the British National Health Service [12]. These data provide consistent findings, demonstrating that similar programs emphasizing carbohydrate restriction as a lifestyle intervention could benefit patients in the United States healthcare system.

At baseline all patients were living with class II or III obesity. This cohort was intentionally selected based on their perceived medical need, and most patients attested to trying diets previously without lasting success. Nevertheless, all 12 patients lost a substantial amount of weight and generally attested to feeling reductions in hunger without feeling deprived. For example, one patient stated, “*I am never hungry anymore, I’ve learned which foods keep me full and which ones don’t. I’ve also found a healthier replacement for everything I couldn’t restrict [in the past], and I don’t feel deprived!*”. While subjective responses were not quantified, there appeared to be a general sentiment that the lifestyle was pleasant and sustainable. Additionally, patients reported being pleased with the knock-on effects that the program had on their family members and loved ones. While the total weight lost across the program patients amounted to 409 lbs (186 kg) in 24 weeks, the patient’s family members reported losing a total of over 200 lbs (91 kg) over this time course, and one patient’s husband who patterned his lifestyle off of that of his wife was able to reverse his type 2 diabetes and discontinue multiple medications. Thus, a theme of these findings, in addition to metabolic health and cost savings, is community. Community within the program presumably helped the patients achieve success, and that success appears to have extended through the family based on anecdotal patient reports.

This study has several limitations. First, only 12 patients were initially enrolled, and only 10 completed the first 24 weeks of the program. The small scale prohibits a demonstration of whether the approach described herein will be scalable. Second, we only report preliminary data from the first 24 weeks of the pilot, and it remains to be seen whether the observed health improvements will be improved upon further, sustained, or regress over time. Third, this is a study with multimodal elements and no control group. Therefore, no firm conclusions can be drawn about the relative efficacy of the approach described herein compared to other dietary lifestyle interventions (although the large effect size of improvements in weight loss and ASCVD risk scores tend to suggest that elements of this model, including carbohydrate restriction, may be particularly efficacious). Fourth, given the individualized nature of the clinic’s approach, the patients’ specific dietary composition were relatively heterogeneous; thus, we make no comment on the relative benefits of different formulations of ketogenic diets. Fifth, we also did not assess the impact of the program on employee work efficiency, which could provide an additional indirect economic benefit; and long-term cost savings may further be underestimated given the high price tag of care for cardiovascular complications [15]. Sixth, we did not formally assess the health improvements that allegedly occurred in the family members of the enrolled patients.

These data also highlight a limitation of the ACC/AHA algorithm. While the patients exhibited a significant 4.04% absolute (44% relative) 10-year reduction for a major adverse cardiovascular event as estimated by the ACC/AHA calculator, these values fail to capture the scope of improvements in cardiovascular risk. Several patients exhibited decreases in total cholesterol and LDL-C during the study, which placed their levels below the minimum for calculator use (as noted above, for these patients, minimum calculator values were entered for 10-year estimates), and the patients exhibited benefits in weight, HbA1c (and HOMA-IR), and CRP that are not captured by the calculator but are relevant to cardiovascular risk.

In conclusion, these data provide preliminary evidence that investment in employee metabolic health by private industry can result in significant improvements in the health of those employees. In terms of future directions, the treatment of this group is ongoing, and due to the success of the program, it is scheduled to be expanded to include more employees. Hopefully, the future will reveal that this program is both scalable and produces lasting results for patient health. Furthermore, in the future, we aim to implement family-style questionnaires to quantify the health impacts on the patients’ family members and to objectively assess the impact of the health improvements observed in patients on their work efficiency and company economics.

## Figures and Tables

**Table 1:** Mean and standard deviations for *n* = 9 patients at baseline, 12, and 24 weeks (5 female, 4 male). * *p* < 0.05, ** *p* < 0.01, *** *p* < 0.001.

	Baseline		12 Weeks (Q1)		24 Weeks		Change	
	Mean	SD	Mean	SD	Mean	SD	Mean	SD
Age	52.9	5.7						
BMI	48.3	6.8	44.5	6.3	41.7	4.8	−6.5 ***	2.9
Weight, lbs (kg)	290.5 (131.8)	44.9 (20.4)	268.0 (121.6)	44.2 (20.2)	252.1 (114.4)	41.1 (18.6)	−38.4 *** (−17.4)	14.8 (6.7)
Fasting glucose	145	45	119	19	110	21	−35 **	35
HbA1C	7.1	1.4	6.4	0.9	6.0	0.6	−1.1 ***	1.4
Total cholesterol	182	32	155	20	175	51	−7	47
HDL-C	44	10	40	8	42	7	−2	6
LDL-C	107	31	97	22	114	49	−6.3	43
Trigs	165	75	110	39	105	36	−60 **	64
Systolic BP	141	13	130	9	124	10	−17 *	17
Diastolic BP	83	7	81	8	78	8	−6	12
ACC/AHA 10-year risk (absolute, %)	9.2	9.7	7.2	6.9	5.2	7.3	−4.0 *	5.6
ACC/AHA 10-year risk (relative change, %)							−44 *	24

## Data Availability

The anonymized raw data from the step-by-step commented code for reproducing both quantitative and graphical analyses are publicly available at: https://github.com/leaduiem/10yrcvdrisk.

## Supplementary Materials

The following supporting information can be downloaded at: https://www.mdpi.com/article/10.3390/metabo12090848/s1, Figure S1: Change in weight, ACC/AHA risk score, and metabolic health markers over 24 weeks; Table S1: Annualized medication cost savings.

## Glossary

ACC	American College of Cardiology
AHA	American Heart Association
CGM	Continuous glucose monitor
CRP	C-reactive protein
HbA1c	glycated hemoglobin
HDL-C	HDL cholesterol
HOMA-IR	homeostatic model of insulin resistance
LDL-C	LDL cholesterol
