# Supplementary material for: A Company Is Only as Healthy as Its Workers: A 6-Month Metabolic Health Management Pilot Program Improves Employee Health and Contributes to Cost Savings
Source: Metabolites. 2022 Sep 9;12(9):848. doi: 10.3390/metabo12090848 (PMC9505533; doi:10.3390/metabo12090848)
Supplement: Supplementary file 1 [file metabolites-12-00848-s001.zip › metabolites-1870822-supplementary.pdf]

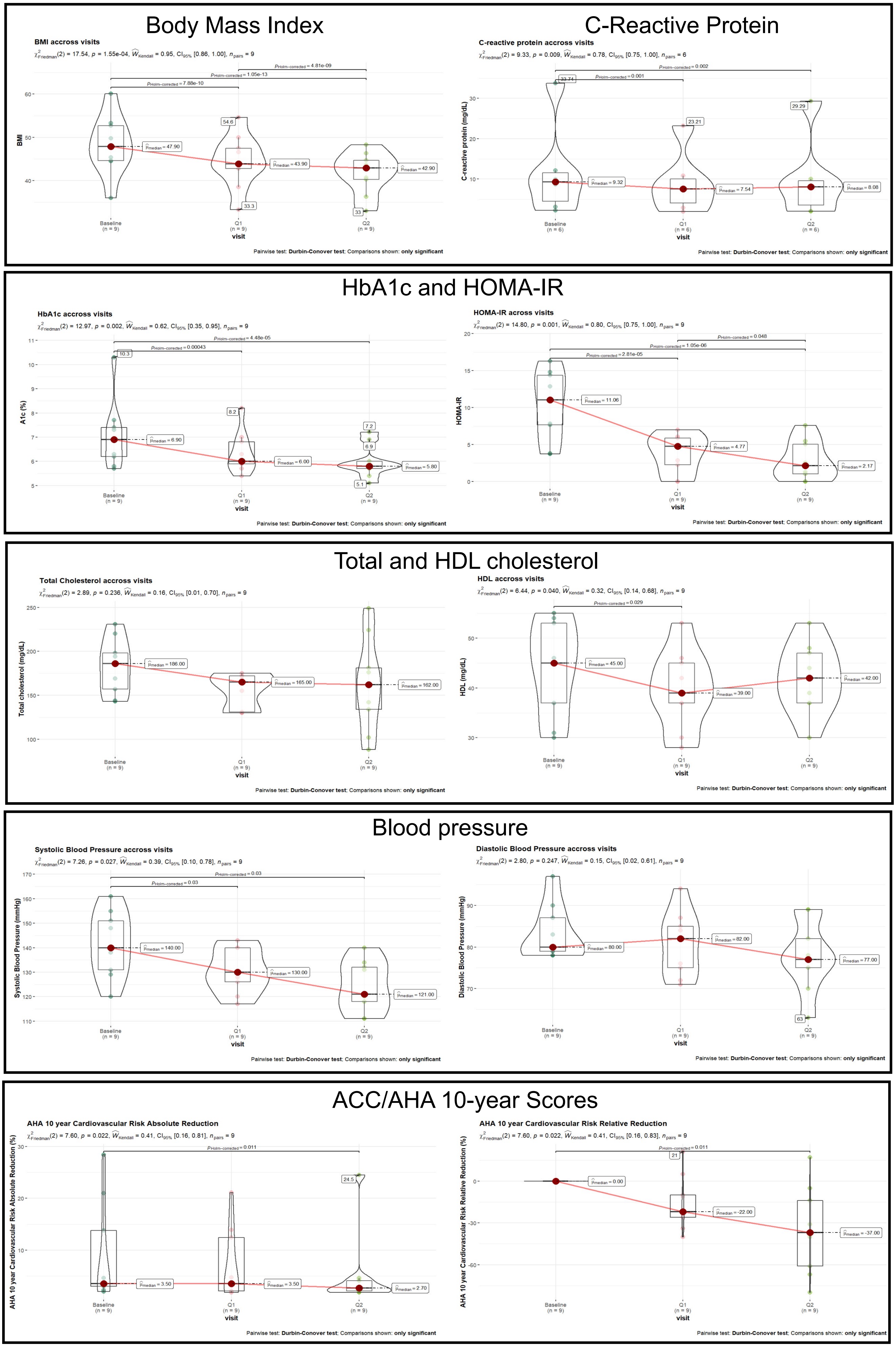

Supplementary Figure S1: Change in weight, ACC/AHA risk score, and metabolic health markers over 24 weeks. Violin box plots with distribution curves were created using the function *ggstatsplot::ggwithinstats*.

			Avg Retail Price Cost Savings	
Medication Name	Quantity	Monthly Cost	Quantity	Annual Cost
Omeprazole 40mg	30 tablets	$64.14	365 tablets	$672.88
Omeprazole 40mg	30 tablets	$64.14	365 tablets	$672.88
Metformin 1000mg	60 tablets	$14.63	730 tablets	$84.80
Metformin 1000mg	60 tablets	$14.63	730 tablets	$84.80
Chlorthalidione 50mg	30 tablets	$33.16	365 tablets	$334.93
Pantoprazole 40mg	30 tablets	$62.50	365 tablets	$521.77
HCTZ 25mg	30 tablets	$8.35	365 tablets	$38.52
HCTZ 25mg	30 tablets	$8.35	365 tablets	$38.52
Sitagliptin 100mg	30 tablets	$520.21	365 tablets	$5,974.15
Sitagliptin 100mg	30 tablets	$520.21	365 tablets	$5,974.15
Insulin Lispro (60units qd)	2 vials/month	$283.41	22 vials/yr	$2,899.51
Insulin Glargine (40units qd)	1 carton/month	$194.29	12 cartons/yr	$2,331.48
Dulaglutide 4.5mg	1 box	$1,029.00	12 boxes/yr	$12,348
Dulaglutide 4.5mg	1 box	$1,029.00	12 boxes/yr	$12,348
Fenofibrate 160mg	30 tablets	$56.00	365 tablets	$460.62
Gemfibrozil 600mg	60 tablets	$27.88	730 tablets	$270.63
Benazepril 40mg	30 tablets	$14.44	365 tablets	$116.06
			Annualized Savings	$45,171.70

Supplementary Table S1: Annualized medication cost savings.
